# Using machine learning to estimate the calendar age based on autonomic cardiovascular function

**DOI:** 10.3389/fnagi.2022.899249

**Published:** 2023-01-23

**Authors:** Andy Schumann, Christian Gaser, Rassoul Sabeghi, P. Christian Schulze, Sven Festag, Cord Spreckelsen, Karl-Jürgen Bär

**Affiliations:** ^1^Lab for Autonomic Neuroscience, Imaging and Cognition (LANIC), Department of Psychosomatic Medicine and Psychotherapy, Jena University Hospital, Jena, Germany; ^2^Hans Berger Department of Neurology, Jena University Hospital, Jena, Germany; ^3^Department of Psychiatry and Psychotherapy, Jena University Hospital, Jena, Germany; ^4^Department of Internal Medicine I, Division of Cardiology, Jena University Hospital, Jena, Germany; ^5^Institute of Medical Statistics, Computer and Data Sciences, Jena University Hospital, Jena, Germany; ^6^SMITH Consortium of the German Medical Informatics Initiative, Leipzig, Germany

**Keywords:** aging, heart rate variability, blood pressure variability, baroreflex, pulse pressure

## Abstract

**Introduction:**

Aging is accompanied by physiological changes in cardiovascular regulation that can be evaluated using a variety of metrics. In this study, we employ machine learning on autonomic cardiovascular indices in order to estimate participants’ age.

**Methods:**

We analyzed a database including resting state electrocardiogram and continuous blood pressure recordings of healthy volunteers. A total of 884 data sets met the inclusion criteria. Data of 72 other participants with an BMI indicating obesity (>30 kg/m^²^) were withheld as an evaluation sample. For all participants, 29 different cardiovascular indices were calculated including heart rate variability, blood pressure variability, baroreflex function, pulse wave dynamics, and QT interval characteristics. Based on cardiovascular indices, sex and device, four different approaches were applied in order to estimate the calendar age of healthy subjects, i.e., relevance vector regression (RVR), Gaussian process regression (GPR), support vector regression (SVR), and linear regression (LR). To estimate age in the obese group, we drew normal-weight controls from the large sample to build a training set and a validation set that had an age distribution similar to the obesity test sample.

**Results:**

In a five-fold cross validation scheme, we found the GPR model to be suited best to estimate calendar age, with a correlation of r=0.81 and a mean absolute error of MAE=5.6 years. In men, the error (MAE=5.4 years) seemed to be lower than that in women (MAE=6.0 years). In comparison to normal-weight subjects, GPR and SVR significantly overestimated the age of obese participants compared with controls. The highest age gap indicated advanced cardiovascular aging by 5.7 years in obese participants.

**Discussion:**

In conclusion, machine learning can be used to estimate age on cardiovascular function in a healthy population when considering previous models of biological aging. The estimated age might serve as a comprehensive and readily interpretable marker of cardiovascular function. Whether it is a useful risk predictor should be investigated in future studies.

## Introduction

Maintaining a healthy cardiovascular system is one of the most important goals of modern health policy (Mendis et al., 2011). Factors elevating cardiovascular risk include physical inactivity and an unhealthy diet. In addition, age is an independent risk factor for the development of cardiovascular disease (CVD)—the leading cause of death worldwide.

The cardiovascular system is a complex structure that comprises the heart and vasculature that are not under voluntary control. Instead, the autonomic nervous system adapts the activity of the heart and vascular tone to changing environmental demands. To assess the state of the cardiovascular system, physicians usually estimate blood pressure and record electrocardiograms (ECGs). Several indicators of cardiovascular risk can be determined from these data.

Heart rate and its variability (HRV) are established markers of cardiac fitness (Jensen et al., 2013; Nanchen et al., 2013). A natural decay in HRV during the course of aging is a consistent finding of several studies (Reardon and Malik, 1996; Fukusaki et al., 2000; Boettger et al., 2010; Voss et al., 2012, 2015). Lower levels of HRV have been associated with increased cardiovascular morbidity and mortality in the elderly (Tsuji et al., 1996). The feedback loop that adapts heart rate to changes in blood pressure, that is, baroreflex function, is progressively diminished with increasing age (Laitinen et al., 2004). Various factors such as endothelial dysfunction or oxidative stress result in the stiffening of large arteries, which is a condition promoting sustained hypertension, atherosclerosis, and thrombosis (Dai et al., 2015). Indicators of age-related vascular changes are broad pulse waves, elevated pulse wave velocity, and increased systolic blood pressure. Considering the widespread effects of aging on the cardiovascular system, it seems useful to combine different established indices into one comprehensive marker of cardiovascular health.

Estimating age based on biological data is a widely used concept in other medical disciplines, for instance, to evaluate brain health (Gialluisi et al., 2019). Aging affects different aspects of brain structure and function that can be summarized as the estimated age of the brain (Franke and Ten Gaser, 2019; Dafflon et al., 2020; Jiang et al., 2020). Using this framework, scientists were able to trace brain development and to assess the risk of developing neurodegenerative diseases and general mortality in older adults (see Cole and Franke, 2017).

Recently, machine learning (ML) methods have gained a lot of attention in efforts to improve risk prediction and clinical outcomes in patients with cardiovascular (see Sevakula et al., 2020, for review). ML algorithms can be used to automatically identify information that will help solve a given problem. Supervised learning methods build an analytical model based on a set of training samples containing input and related output values. Applying this model to a test set of input data without knowing the desired output reveals the accuracy of the automatic solution. For regression problems, an output function is obtained by fitting a line to the data points in a high-dimensional space built from available input variables (feature space; Bennett and Campbell, 2000; Schölkopf and Smola, 2002). Assessing cardiovascular risk by ML has been demonstrated to be more accurate than conventional approaches (Kakadiaris et al., 2018) with a lower bias than non-ML methods (Suri et al., 2022).

In this study, we aimed to estimate age based on cardiovascular data by applying ML. Input features were extracted from simultaneous resting recordings of ECG and continuous blood pressure in healthy individuals. We compared different approaches to solve regression problems, namely, support vector regression, relevance vector regression, Gaussian process regression, and a linear regression model. In a proof-of-concept application, we compared age estimates in obese but otherwise healthy individuals and normal-weight controls. As obesity is related to an impairment of cardiovascular function and elevated cardiovascular risk, we assumed systematically higher age estimates when compared to normal-weight controls. Thus, we derived three age-matched subsamples from our database. We trained all ML models on normal-weight controls and applied them to a sample of obese individuals and an independent sample of normal-weight controls.

## Materials and methods

### Database

Resting-state physiological recordings of 1,121 healthy volunteers were obtained. None of the subjects had any history of neurological or psychiatric disorders. Exclusion criteria were any medical conditions, illegal drugs, or medication potentially influencing cardiovascular function. Thorough physical examination, resting electrocardiography (ECG), and routine laboratory parameters (electrolytes, basic metabolic panel, and blood count) had to be without any pathological finding. All participants provided written informed consent before participating in the study. The study protocol was approved by the Ethics Committee of the University Hospital of Jena (#5423-01/18, 4,940-10/16). Data sets have been made publicly available at *PhysioNet* (Goldberger et al., 2000; Schumann and Bär, 2022).

From the original database, 31 subjects were excluded due to missing or invalid information regarding age, gender, or BMI. A total of 118 recordings were excluded due to incomplete or missing blood pressure recordings. A set comprising data from 72 obese participants (body mass index (BMI) > 30) was excluded from the main set but used for additional assessments during a later stage. Cardiovascular indices were estimated for the 884 healthy subjects from the main set (59% females, age: 31.3 ± 13.6 years, BMI: 22.8 ± 2.8 kg/m^2^, see Table 1) and for the 72 obese participants.

**Table 1:** Sample characteristics separated by recording device.

	TFM	MP150
Age [y]	31.9 ± 14.3	30.5 ± 12.6
*N*	18–92	18–82
Sex (f/m)	283/205	240/156
BMI [kg/m^2^]	22.7 ± 2.9	22.8 ± 2.7

Age and BMI are given in mean value ± standard deviation. Data have been recorded using two different devices (see the Data Recordings section, for more details). TFM, Recorded by CNSystems Task Force Monitor; MP150, Recorded by BIOPAC Systems MP150; BMI, body mass index.

### Data recordings

Continuous non-invasive blood pressure and ECG were acquired simultaneously over 20 min in the supine position using either a Task Force Monitor® (TFM, CNSystems Medizintechnik GmbH, Graz, Austria) or MP150 (BIOPAC Systems Inc., Goleta, CA, United States). First 5 min were excluded from the analysis. R-waves and systolic and diastolic blood pressure values were extracted from the data using automatic detection algorithms delivered with the devices (Task Force® Monitor, CNSystems or AcqKnowledge 4.1, BIOPAC Systems). An adaptive filter procedure was applied to identify and substitute premature ventricular beats and artifacts based on the heart beat intervals (Wessel et al., 2000). Data sets with an artifact rate larger than 5% of all intervals were excluded from the analysis.

### Estimation of cardiovascular indices

From the ECG-derived heart beat interval time series (BBI), we calculated the mean heart rate (HR), root-mean-square of successive BBI (RMSSD), the standard deviation of BBI (SDNN), low- and high-frequency power and their ratio (Malik et al., 1996), deceleration capacity (Bauer et al., 2006), Renyi entropy (base 1/4; Renyi, 1961), sample entropy (Richman and Moorman, 2000), and compression entropy (Baumert et al., 2004). The mean and standard deviation of corrected QT intervals (Hodges et al., 1983) and the QT variability index (QTVI) were estimated (Berger, 2003).

From continuous blood pressure, the mean and standard deviation of systolic blood pressure (SBP) and diastolic blood pressure (DBP) values per heart beat interval were extracted (Floras, 2013). Pulse pressure was calculated as differences between SBP and DBP. Using the dual sequence method, baroreflex sensitivity was calculated as a marker of bradycardic and tachycardic changes due to blood pressure alterations (Malberg et al., 1999). Mean values and standard deviation of the pulse transit time, pulse rise time, pulse wave duration, pulse wave velocity, and time delay of the dicrotic notch were estimated on blood pressure signals (Fischer et al., 2017; Table 2).

**Table 2:** Indices included in age estimation.

Index	Explanation
**Standard heart rate variability (HRV)**	
HR [min^−1^]	Mean heart rate
SDNN [ms]	Standard deviation of heart beat intervals (BBI)
RMSSD [ms]	Root-mean-square of successive BBI differences
DC [ms]	Deceleration capacity
**Spectral HRV**	
LF [ms^2^]	Low frequency spectral power of BBI
HF [ms^2^]	High-frequency spectral power of BBI
LF/HF [a.u.]	Low-to-high frequency spectral power ratio
**Nonlinear HRV**	
CompEn [a.u.]	Compression entropy
SampEn [a.u.]	Sample entropy
RenyiEn [bit]	Renyi entropy
**Cardiovascular regulation**	
BRS [ms/mmHg]	Baroreflex sensitivity
LFalpha [ms/mmHg]	Low frequency cardiovascular coherence
HFalpha [ms/mmHg]	High-frequency cardiovascular coherence
JSDsym [a.u.]	Symmetric joint symbolic dynamics
SBP [mmHg]	Mean systolic blood pressure
sd_SBP [mmHg]	Standard deviation of systolic blood pressure
DBP [mmHg]	Mean diastolic blood pressure
sd_DBP [mmHg]	Standard deviation of diastolic blood pressure
PP [mmHg]	Pulse pressure
**Pulse wave dynamics**	
PTT [ms]	Mean pulse transit time
sdPTT [ms]	Standard deviation of PTT
PRT [ms]	Mean pulse rise time
sdPRT [ms]	Standard deviation of PRT
PWV [mmHg/ms]	Mean pulse wave velocity
sdPWV [mmHg/ms]	Standard deviation of PWV
SIT [ms]	Mean delay of dicrotic notch to pulse maximum
**QT interval characteristics**	
meanQTc [ms]	Mean corrected QT interval
sdQTc [ms]	Standard deviation of QT interval
QTVI [a.u.]	QT variability index
**Additional info**	
Sex [0/1]	Participant gender
Device [0/1]	Recording device

### Age estimation

Four different ML approaches were applied to estimate calendar age based on 29 cardiovascular indices and the two categorial variables sex and recording device. The algorithms have been implemented in Python version 3.8.3 using the toolbox scikit-learn version 0.24.1 (Pedregosa et al., 2011).

**Gaussian process regression (GPR)** models use a kernel to define the covariance of a distribution over the target functions and observed training data to define a likelihood function (Schulz et al., 2018). We used a combination of a constant kernel with a radial basis function (RBF). **Support vector regression (SVR)** models offer the flexibility to define how much error is acceptable in finding an appropriate fit to the input data (Vapnik, 1995; Schölkopf and Smola, 2002). An RBF kernel and regularization index C = 30 were used. **Relevance vector regression (RVR)** models use Bayesian inference to obtain parsimonious solutions for regression (Tipping, 2000). Here, we also used an RBF kernel. Hyperparameters of GPR, SVR, and RVR estimation were optimized using grid search. The performance of these approaches was compared to a **linear regression (LR)** model that estimates age as a linear combination of the input variables.

### Model performance

In a 5-fold cross-validation scheme, one-fifth of the main set was randomly assigned to a test set, while the model was trained on the remaining four-fifths of the data. In each of the five runs, another fifth of the data served as test data. After the five runs, the empiric means (± standard error) of the evaluation metrics are reported as the final ones. The cross-validation was repeated 20 times with a randomized order of input data. Again, the metrics were averaged over all repetitions. We standardized all input data before using training and test data during the cross-validation procedure (*StandardScaler* implemented in *sklearn*).

The quality of age estimation was evaluated by the mean absolute error (MAE), root-mean-squared error (RMSE), and Pearson’s correlation (r) of estimated $\overset{\wedge }{Y_{i}}$ and the actual age *y_i_*.


$$MAE=\frac{\sum_{i}\left(\left|{\overset{\wedge }{Y}}_{i}-Y_{i}\right|\right)}{N}$$

$$RMSE=\sqrt{\frac{\sum_{i}{\left(\overset{\wedge }{Y_{i}}-Y_{i}\right)}^{2}}{N}}$$

### Comparison of weight groups

In a second experiment, we aimed to compare age estimates in obese participants (BMI > 30 kg/m^2^) with normal-weight peers. Therefore, we draw two subsamples from our normal-weight population that served as training and test data. The calendar age of these data should match our obese sample. Therefore, we categorized participants into age groups of 10 years (see Table 3) and estimated the relative distribution of obese participants across these age groups. Then, we randomly assigned normal-weight participants from each age group to training and a test set to match the age distribution of the obese sample. Finally, we trained all ML models on 197 normal-weight individuals (101 women, 96 men, 41.4 ± 14.9 years, BMI: 23.4 ± 2.2 kg/m^2^) to estimate age in the obese test sample of 72 individuals (44 women, 28 men, age: 42.9 ± 15.5 years, BMI: 34.8 ± 5.8 kg/m^2^) in a normal test sample of 72 normal-weight controls (37 women, 35 men, age: 42.1 ± 15.8 years, BMI: 23.3 ± 2.4 kg/m^2^). We used mean values and standard deviation of training data to standardize the training, normal and obese test sets (*StandardScaler*, *sklearn*). The age gap (deviation between estimated and calendar age) was calculated and compared between the normal-weight and the obese test set using the one-sided Wilcoxon rank-sum test.

**Table 3:** Sample description in different age ranges.

	Healthy participants in age groups				
	<30 years	30–39 years	40–49 years	50–59 years	≥60 years
Age [y]	23.7 ± 2.7	33.6 ± 2.9	44.4 ± 2.8	53.5 ± 2.7	70.1 ± 9
*N*	585	123	75	47	54
Sex (f/m)	386/199	54/69	30/45	16/31	38/16
BMI [kg/m^2^]	22.1 ± 2.6	23.2 ± 2.7	24.3 ± 2.9	25.2 ± 2.7	24.8 ± 2.5

Results are given in mean values ± standard deviation. BMI: body mass index.

## Results

The final sample under investigation included 884 healthy individuals. In Table 3, sample characteristics are depicted within different age ranges.

**Figure 1:**
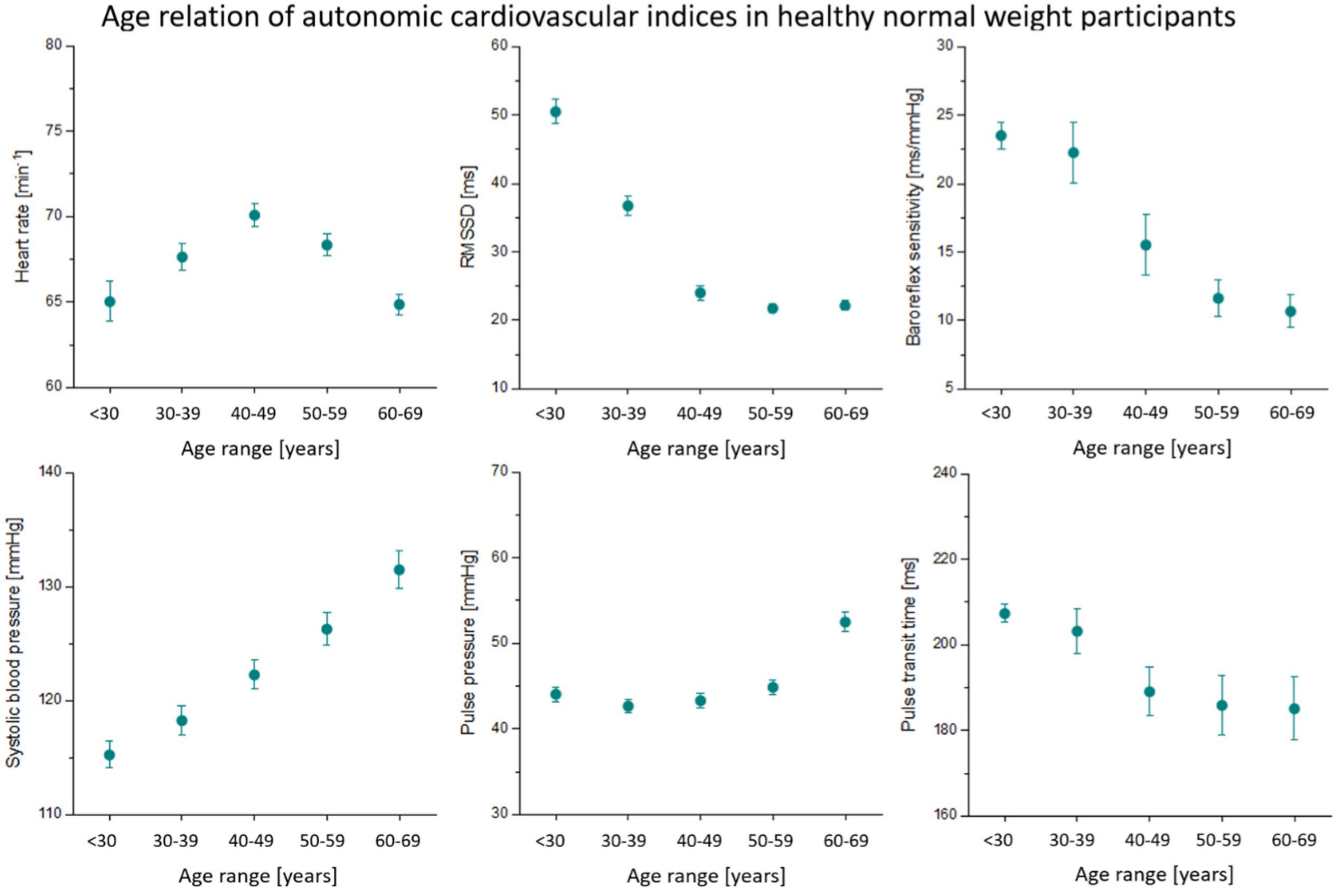
Age dependency of autonomic cardiovascular indices. Mean values and 95% confidence intervals are depicted. RMSSD, vagal heart rate variability (root-mean-square of successive heart beat intervals).

A number of relevant autonomic cardiovascular indices are depicted in Figure 1. It seems obvious from these plots that age has a different effect on each of those measures. For instance, systolic blood pressure seems to increase almost linearly with age, while HRV decreases with age rather exponentially. In total, 29 different indices together with *sex* and *device* served as input features for age prediction models.

**Figure 2:**
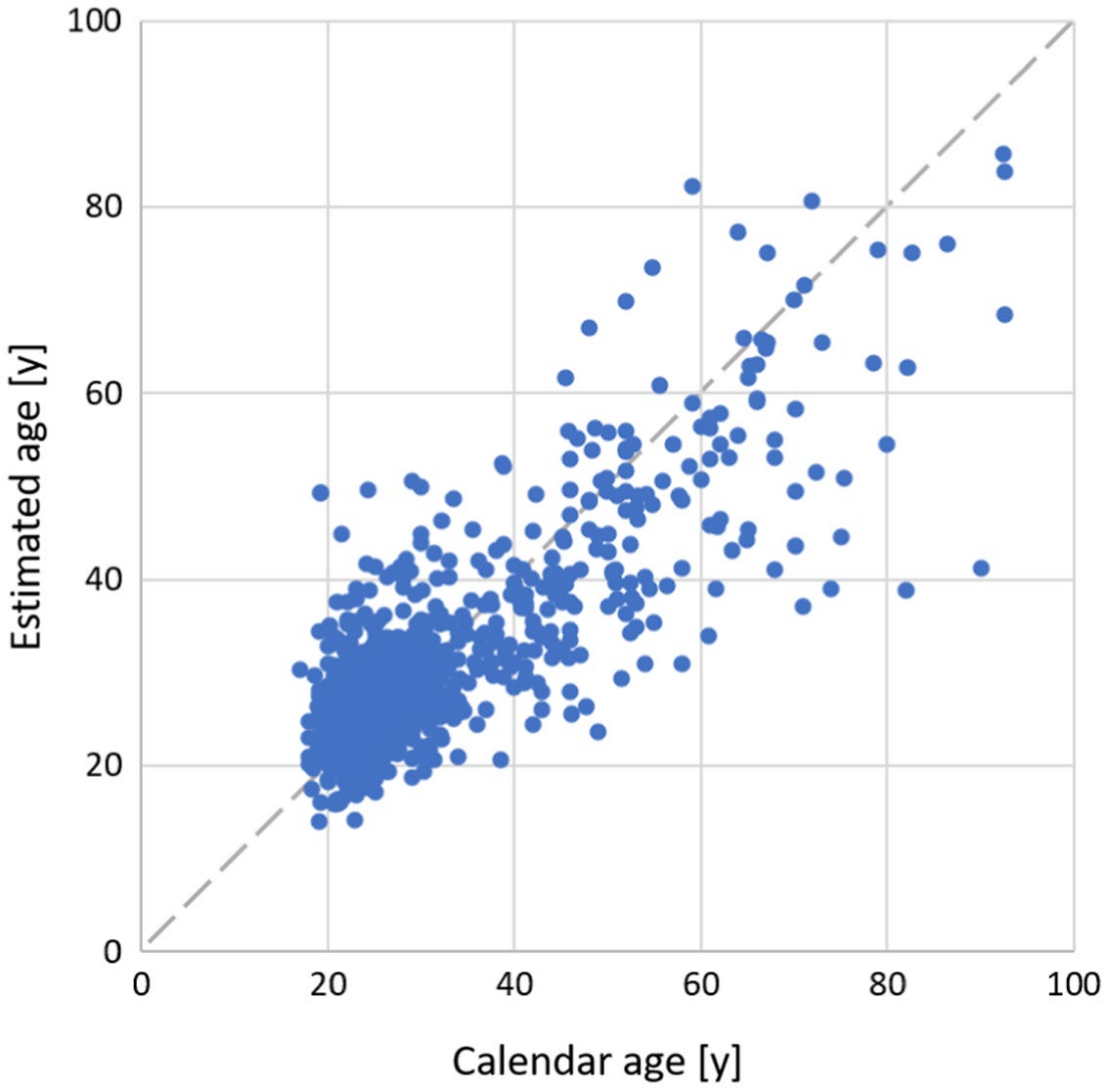
Relation of individual age estimated by GPR and calendar age. Dashed gray line indicates perfect concordance.

In Figure 2, we plotted the age estimated by Gaussian process regression (GPR) against the calendar age for one cross-validation run (Figure 2; r = 0.81, MAE = 5.62 years, RMSE = 8.00 years). In this scatter plot, it becomes clear that most of the data sets are in the lower age range. At a higher age (over 70 years), the model tends to underestimate the individual age.

### Comparison of methods

After 20 runs of the 5-fold cross-validation, we compared performance measures of age prediction by four different mathematical models (see Table 4). Lowest error estimates were achieved using GPR to estimate age (MAE = 5.6 years and RMSE = 8.0 years). The highest correlation was also calculated between GPR estimates and underlying calendar age. Overall performance tended to be better in men compared to women. However, in women and men, GPR was the most accurate approach to estimate age in terms of errors and correlation to underlying calendar age.

**Table 4:** Performance scores of age prediction after 20 repetitions of cross-validation.

Performance scores	Regression approach			
	LR	GPR	SVR	RVR
MAE [y]	6.39 ± 0.36	**5.64 ± 0.33**	6.45 ± 0.33	6.15 ± 0.8
RMSE [y]	8.67 ± 0.06	**8.03 ± 0.08**	8.86 ± 0.13	8.71 ± 0.27
r	0.77 ± 0.01	**0.81 ± 0.01**	0.77 ± 0.01	0.77 ± 0.01
**Performance scores in women (*N* = 523)**				
MAE [y]	6.47 ± 0.06	**6.01 ± 0.1**	6.96 ± 0.15	6.5 ± 0.3
RMSE [y]	8.53 ± 0.09	**8.37 ± 0.13**	9.48 ± 0.2	9.06 ± 0.37
r	0.73 ± 0.01	**0.74 ± 0.01**	0.68 ± 0.01	0.7 ± 0.02
**Performance scores in men (*N* = 361)**				
MAE [y]	6.33 ± 0.05	**5.38 ± 0.06**	6.09 ± 0.1	5.91 ± 0.23
RMSE [y]	8.76 ± 0.08	**7.79 ± 0.09**	8.4 ± 0.13	8.46 ± 0.27
r	0.79 ± 0.01	**0.83 ± 0.01**	0.81 ± 0.01	0.8 ± 0.01

The lowest error estimates and highest correlation coefficient are written in bold. LR, linear regression; GPR, Gaussian process regression; SVR, support vector regression; RVR, relevance vector regression; r, Pearson’s correlation coefficient; MAE, mean absolute error; RMSE, root-mean-squared error.

### Age gap estimation

**Figure 3:**
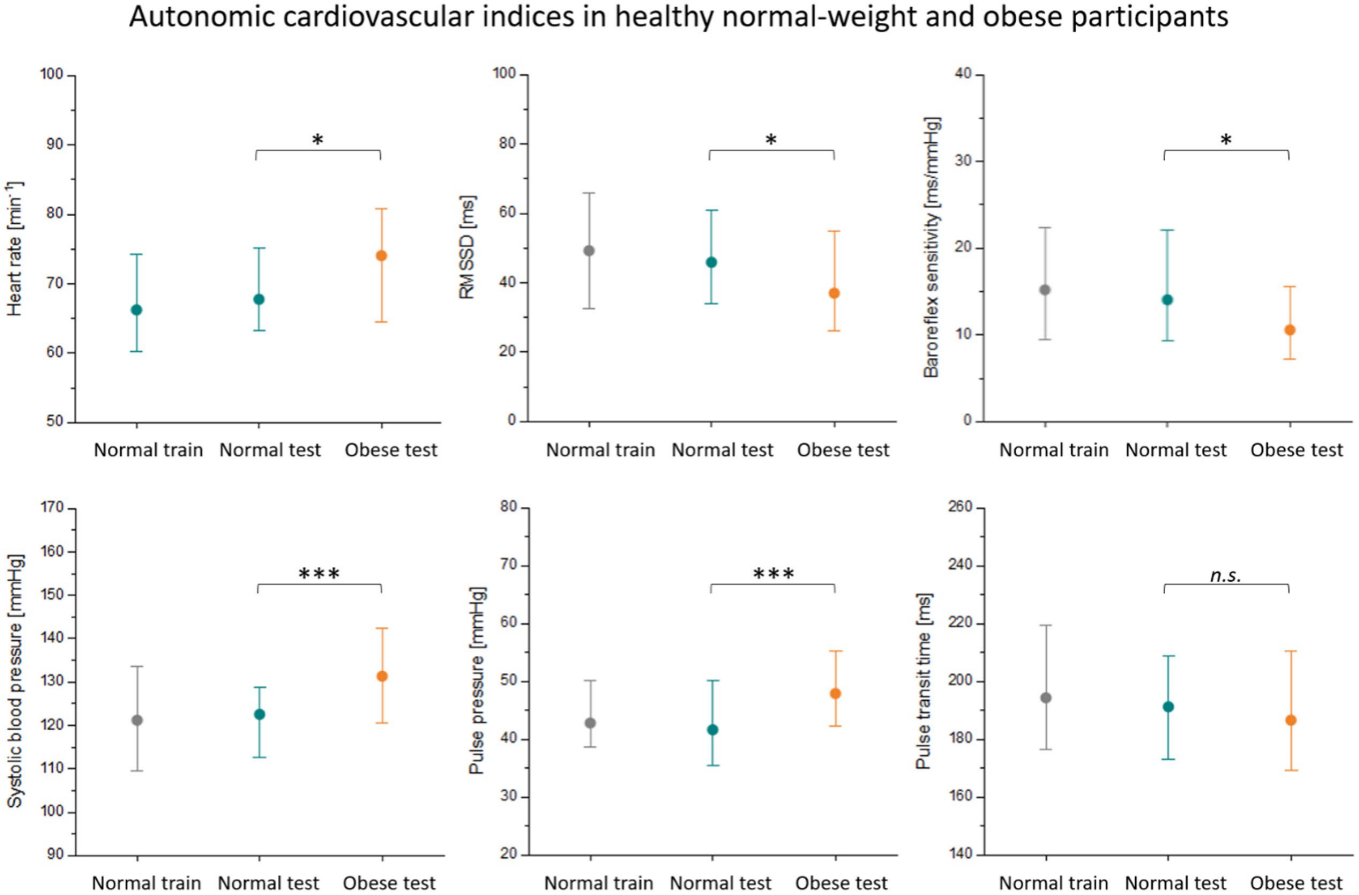
Autonomic cardiovascular indices in two subsets of normal-weight participants (training data in gray, test data in cyan) and obese participants (orange). The median is depicted together with the lower quartile and the upper quartile. The two test sets were compared using the Wilcoxon rank-sum test with *p*-values indicated in the figure [*p* < 0.05 (*); *p* < 0.001 (***); *p* > 0.05 (n.s.)]. RMSSD, vagal heart rate variability (root-mean-square of successive heart beat intervals).

Figure 3 shows the estimated autonomic indices for the normal-weight train group (normal train), the normal-weight test group (normal test), and the obese test group (obese test). Results from the group comparison between the two test sets are shown in Supplementary Table S1. Compared to normal participants, obese individuals showed alterations in a number of autonomic indices, including elevated heart rates, reduced vagal heart rate variability (RMSSD) and baroreflex sensitivity, and increased blood pressure (see Figure 3). The two normal-weight groups did not seem to differ significantly from one another except for the probability of symmetric symbolic dynamics of blood pressure and heart rate (JSDsym, see Supplementary Table S1).

**Figure 4:**
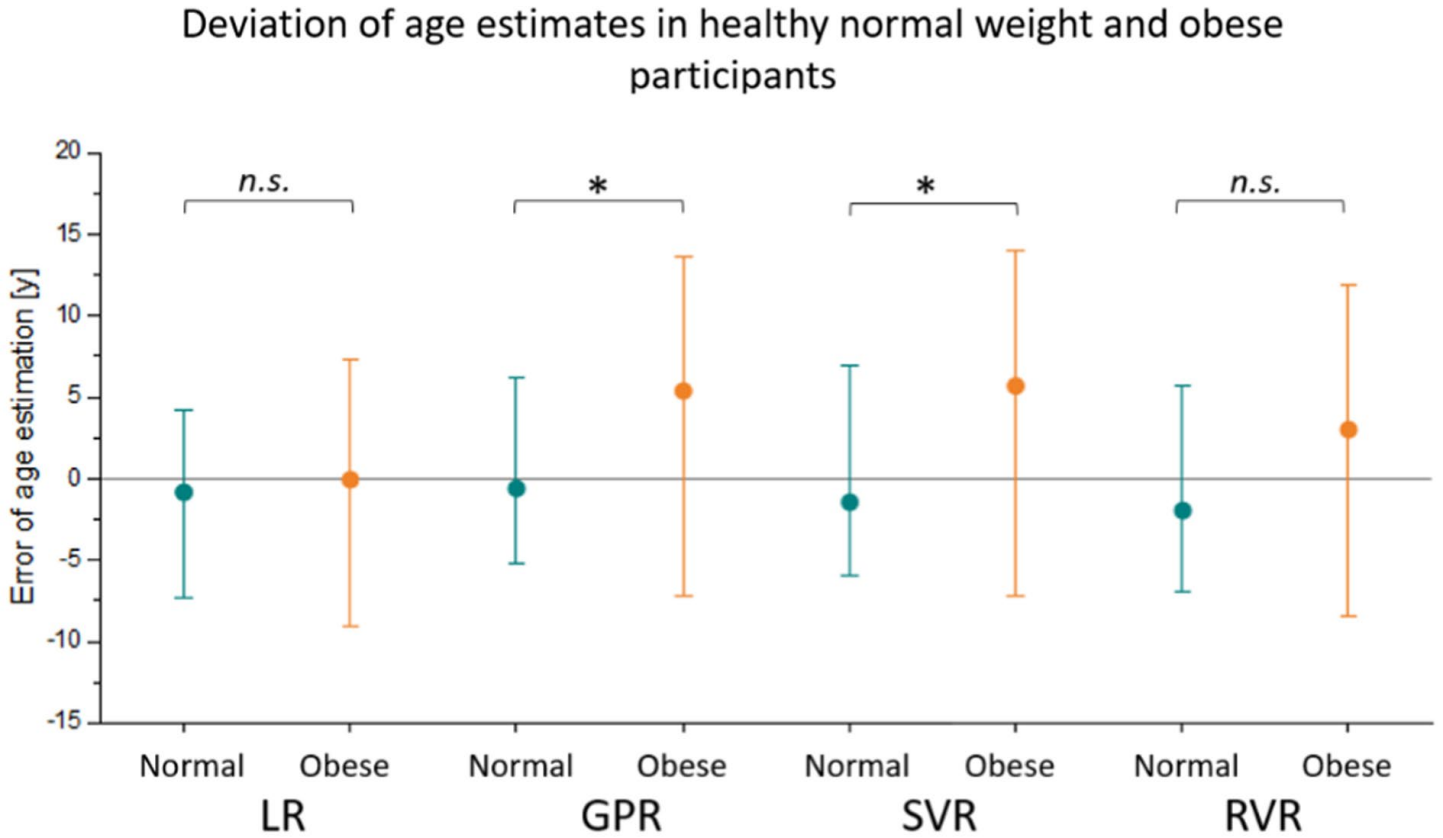
Deviation of estimated age from calendar age in a sample of obese participants (orange) and an independent set of matched normal-weight controls (cyan). The median is depicted together with the lower quartile and the upper quartile. Four different models, namely linear regression (LR), Gaussian process regression (GPR), support vector regression (SVR), and relevance vector regression (RVR), were trained on matched normal-weight individuals. The age estimation errors were compared between the normal-weight and obese test set using the Wilcoxon rank-sum test with p-values indicated in the figure [*p* < 0.05 (*); *p* > 0.05 (n.s.)].

All four methods were used to estimate the age gap between normal-weight healthy individuals (*N* = 72) and obese but apparently healthy individuals (*N* = 72). In Figure 4, deviations between calendar age and ML estimates per method are illustrated. The average errors of age estimation were higher in obese participants for all four methods. The Wilcoxon rank-sum test revealed a significantly increased age estimation error in obese participants by 5.4 years (interquartile range IQR = [−7.2; 13.1]; z = −1.744, *p* = 0.0406) when using GPR and 5.7 years (IQR = [−7.2;13.6]; z = −2.148; *p* = 0.0159) when using SVR.

## Discussion

As artificial intelligence has entered most aspects of our daily life, it is not surprising that machine learning (ML) is about to revolutionize the medical sector and healthcare industry (Chen et al., 2017; Koch, 2018). ML offers great opportunities to improve risk stratification, diagnostic classification, clustering for the identification of patient subgroups, and many more (Sevakula et al., 2020). One popular application is the quantification of aging effects based on biological information (Al Zoubi et al., 2018; Dafflon et al., 2020; Jiang et al., 2020). To the best of our knowledge, this is the first study to use autonomic markers from cardiovascular recordings to predict chronological age in healthy subjects using ML.

Estimated age was strongly correlated with actual age with an error of MAE = 5.6 years, RMSE = 8.0 years, and r = 0.81 when GPR was used. However, the accuracy of age estimation was lower in older participants. For instance, age was underestimated in participants over 70 years of age by the GPR model (Figure 2). As data sets used in our study are concentrated at a younger age (<40 years), models are primarily trained on young individuals. This makes the prediction of age more difficult in the elderly. The accuracy of the models was higher in men than in women. This might be due to the fact that some age-related changes are more pronounced in men than in women. Especially, blood pressure and vascular indicators of arterial stiffening have been reported to correlate stronger with age in men (AlGhatrif et al., 2013). Maybe an additional influence of the menstrual cycle may have increased the variance of estimated features in women that we were not able to account for (Schmalenberger et al., 2020).

Considering state-of-the-art approaches to estimate age based on biological information, as reviewed by Gialluisi et al. (2019), the accuracy of our models was quite high. According to their summary, brain data and blood markers have been more widely used to estimate age, with MAEs ranging between 4.2 and 11.8 years. Only five of the 14 studies that have been reviewed achieved MAEs below 5 years—all based on brain data. Analyzing blood values, the most accurate model had an MAE of 5.6 years. In contrast, ML approaches to evaluate the aging of the cardiovascular system are rare. Using linear regression models, few studies have already attempted to estimate age based on HRV and ECG. Colosimo (1997) used a linear model to estimate age that correlated with calendar age with r = 0.71 in 141 subjects. More recently, Starc et al. (2012) predicted age using HRV and a multiple linear regression model with a high correlation of r = 0.87 in 377 subjects. Unlike those approaches, ML techniques automatically determine a numerical solution from a variety of input data through the learning process. Input features can be of different types (scalar values, signals, and images), and finally, they can contribute in a nonlinear fashion to this solution. Therefore, ML strategies often improve the accuracy of mathematical models in several applications (e.g., Acevedo et al., 2009; Ren et al., 2020).

In this study, we used a variety of established cardiovascular indices as input features. However, there are countless measures of heart rate variability alone. Instead of calculating these variables on physiological recordings, the recorded signals themselves can be entered into the models. Relevant signal segments then contribute to the estimation of age, avoiding the selection of suitable cardiovascular indices. *Via* methods of deep learning, Attia et al. (2019) estimated the calendar age from short 12-lead ECG signals. A convolutional neural network led to an error of MAE = 6.9 years and r = 0.84. Those patients, whose predicted age was more than 7 years higher than their calendar age, were more likely to be diagnosed with cardiovascular diseases, such as hypertension or coronary disease. The authors acknowledged that one key limitation to the findings in their study was the fact that the large underlying sample of 774,783 subjects included only patients who had their ECG recorded for some clinical indication. Similarly, Strodthoff et al. (2021) estimated calendar age based on short 12-lead ECG records using different neural networks. For age estimation, a feedforward residual neural network performed best with an error of MAE = 6.86 years and r = 0.85. Their database included a total of 21,837 both normal and abnormal clinical ECG recordings of 18,885 patients.

In our study, we estimated the deviation from normal healthy aging in a sample of obese but otherwise healthy participants as proof-of-concept. All four models were trained on normal-weight controls and then used to estimate age in a normal-weight and an obese test set. Calendar age distribution was matched across subsets. Using GPR and SVR, the age gap between estimated age and calendar age was significantly higher in obese participants than in normal-weight controls. This means that there was a systematic overestimation of age in obese participants. At least some differences in cardiovascular indices between obese and normal-weight participants are similar to changes that occur during aging. We observed elevated systolic blood pressure and pulse pressure as well as lower vagal HRV and baroreflex sensitivity in obese individuals and in older age groups of normal-weight participants (Figure 3). These alterations are signs of arterial stiffening and a loss of cardiovagal control that can be observed in elderly individuals (Pinto, 2007). Advanced cardiovascular aging was suggested by an age gap over 5 years when compared to matched normal-weight controls.

The relationship between body mass index (BMI) and mortality is well documented (see review by Aune et al., 2016). While increased BMI raises mortality risk (Chen et al., 2019), a large population-based study has recently demonstrated that weight loss can prevent premature death in later life (Xie et al., 2020). Participants who reduced their BMIs below the obese range between early adulthood through midlife halved their mortality risk compared with those remaining obese, suggesting that the physiological effects of obesity may be reversible to some extent. Expressing cardiovascular impairment in terms of advanced age might help to convince those individuals at risk to adopt a healthier lifestyle (Cuende, 2016).

## Limitations

The current study relies on physically and mentally healthy subjects who were recruited for resting physiological recordings under standardized conditions. However, the size of the sample is, therefore, rather small. Although we investigated over a thousand subjects, the number of data sets actually included in the analysis was reduced by quality control. Especially at older ages, a rather small amount of data was available. The recruitment of participants of an advanced age without being affected by cardiovascular, neurological, or psychiatric disorders is very complicated. Further cognitive impairment, sensory loss, and changes in mobility might introduce a selection bias (Young and Vitaliano, 2006).

Another limitation of our database is that there is no information on general health in order to account for it in our analysis, such as metabolic markers, smoking or drinking habits, or mental health. Because we also lack longitudinal data, we are unable to evaluate how autonomic status continues to change. Aging of the cardiovascular system is, most probably, not an entirely linear process. Intercurrent life events might moderate the rate of age-related changes. An intriguing line of further research is to assess the potential of the estimated age to predict cardiovascular risk.

## Conclusion

In this study, we estimated age based on autonomic cardiovascular indices with high accuracy in healthy controls. The Gaussian process regression model led to the best concordance of estimated and calendar age. Using this framework, it seems possible to quantify deviations from healthy autonomic aging. In this study, cardiovascular changes in obese but otherwise healthy individuals led to an advanced age of more than 5 years compared with normal-weight controls. In future studies, the clinical value of the gap between the individual calendar and the estimated autonomic age to indicate diseases of the circulatory system or its potential to predict cardiovascular risk needs to be explored.

## Data availability statement

Publicly available datasets were analyzed in this study. This data can be found at: https://physionet.org/content/autonomic-aging-card; https://doi.org/10.13026/2hsy-t491

## Ethics statement

The studies involving human participants were reviewed and approved by Ethics Committee of the University Hospital of Jena. The patients/participants provided their written informed consent to participate in this study.

## Author contributions

AS performed data analysis and wrote the manuscript. CG supervised analysis and interpretation of data. PCS advised the interpretation of results and critically revised the manuscript. RS, SF, and CS supervised analysis and critically revised the manuscript. K-JB conceived the study and prepared and critically revised the manuscript. All authors contributed to the article and approved the submitted version.

## Funding

This research was funded by the German Research Foundation (DFG, BA 3848/9-1 and SCHU 3432/2-1) and the Interdisciplinary Centre for Clinical Research Jena (IZKF, MSP05-2019).

## Conflict of interest

The authors declare that the research was conducted in the absence of any commercial or financial relationships that could be construed as a potential conflict of interest.

## Publisher’s note

All claims expressed in this article are solely those of the authors and do not necessarily represent those of their affiliated organizations, or those of the publisher, the editors and the reviewers. Any product that may be evaluated in this article, or claim that may be made by its manufacturer, is not guaranteed or endorsed by the publisher.
