# Supplementary material for: Using machine learning to estimate the calendar age based on autonomic cardiovascular function
Source: Front Aging Neurosci. 2023 Jan 23;14:899249. doi: 10.3389/fnagi.2022.899249 (PMC9899796; doi:10.3389/fnagi.2022.899249)
Supplement: Supplementary file 1 [file Table_1.pdf]

Supplementary Table S1 Autonomic cardiovascular indices in two subsets of normal-weight participants (training data, test data) and obese participants given as median and quartiles [25%, 75%]. The group comparison of the obese and normal-weight test set was conducted using the Wilcoxon rank-sum test (right column).

Index	Normal train	Normal test	Obese Test	Significance
HR [min-1]	66.3 [60.2;74.5]	67.8 [63.2;75.2]	74.1 [64.5;80.7]	z=-2.156; p=0.031
SDNN [ms]	32.2 [19.9;50.7]	32.3 [18.2;51.6]	24.4 [16.1;44.7]	z=1.201; p=0.230
RMSSD [ms]	49.3 [32.6;65.8]	46.0 [34.0;60.8]	37.0 [26.3;55.0]	z=2.044; p=0.041
DC [ms]	8.79 [6.52;11.13]	7.35 [5.89;10.16]	6.26 [4.79;9.05]	z=2.164; p=0.030
LF [ms2]	1088 [511;2109]	878 [585;1519]	704 [353;1385]	z=1.580; p=0.114
HF [ms2]	818 [419;1743]	808 [358;1569]	499 [205;1173]	z=1.656; p=0.098
LF/HF [a.u.]	1.87 [1.15;3.13]	1.89 [1.07;4.24]	2.09 [1.07;3.57]	z=0.497; p=0.619
CompEn [a.u.]	0.74 [0.68;0.8]	0.74 [0.66;0.82]	0.70 [0.62;0.76]	z=2.847; p=0.004
SampEn [a.u.]	2.17 [1.86;2.61]	2.25 [1.79;2.72]	2.03 [1.8;2.41]	z=1.560; p=0.119
RenyiEn [bit]	2.91 [2.53;3.28]	2.98 [2.47;3.36]	2.76 [2.45;3.12]	z=1.660; p=0.097
BRS [ms/mmHg]	15.2 [9.5;22.5]	14.1 [9.3;22.1]	10.6 [7.2;15.6]	z=2.188; p=0.029
LFalpha [ms/mmHg]	10.2 [6.4;15.3]	9.5 [5.9;11.9]	8.3 [5.4;12.2]	z=0.833; p=0.405
HFalpha [ms/mmHg]	11.4 [7.9;19.7]	11.7 [6.5;15.8]	9.6 [6.6;15.3]	z=0.917; p=0.359
JSDsym [a.u.]	0.17 [0.09;0.34]	0.27 [0.14;0.37]	0.26 [0.11;0.38]	z=0.222; p=0.825
SBP [mmHg]	121 [109;134]	123 [113;129]	131 [121;142]	z=-3.734; p<0.001
sd_SBP [mmHg]	4.76 [3.63;6.56]	5.14 [4.47;6.7]	4.79 [3.76;6.65]	z=1.237; p=0.216
DBP [mmHg]	75 [67;85]	77 [71;86]	82 [72;90]	z=-1.956; p=0.050
sd_DBP [mmHg]	3.58 [2.75;4.71]	3.97 [3.06;4.46]	3.51 [2.65;4.87]	z=1.209; p=0.227
PP [mmHg]	42.9 [38.7;50.3]	41.7 [35.6;50.1]	48.0 [42.2;55.3]	z=-3.680; p<0.001
PTT [ms]	194 [176;220]	191 [173;209]	187 [169;211]	z=0.394; p=0.694
sdPTT [ms]	13.7 [7.2;22.3]	12.0 [5.5;19.6]	12.0 [5.3;14.7]	z=0.57; p=0.566
PRT [ms]	116 [106;136]	112 [104;123]	111 [102;120]	z=0.813; p=0.416
sdPRT [ms]	12.6 [9.5;16.5]	12.6 [8.0;17.4]	10.3 [6.8;15.7]	z=1.320; p=0.187
PWV [mmHg/ms]	261 [232;294]	248 [227;278]	244 [226;288]	z=0.238; p=0.812
sdPWV [mmHg/ms]	17.93[6.5;22.2]	10.5 [5.7;20.0]	10.8 [5.8;21.6]	z=-0.420; p=0.673
SIT [ms]	220 [200;237]	221 [205;234]	226 [210;249]	z=-1.938; p=0.053
meanQTc [ms]	0.42 [0.39;0.46]	0.43 [0.4;0.47]	0.45 [0.41;0.48]	z=-1.688; p=0.091
sdQTc [ms]	0.024 [0.018;0.032]	0.022 [0.016;0.029]	0.022 [0.016;0.03]	z=0.010; p=0.992
QTVI [a.u.]	-1.19 [-1.49;-0.54]	-1.12 [-1.37;-0.77]	-0.89 [-1.33;-0.37]	z=-2.156; p=0.031
